# Progress in Surgery

**Published:** 1896-06-13

**Authors:** 


### Progress in Surgery.

### GENITO-URINARY SURGERY.

**Enlarged Prostate.**—Mr. Mansell Moullin[1] calls attention to the fact that some men make use of catheters for years without taking any precautions and do not get cystitis, but others carry into the bladder organisms which abound in the urethra and soon set up this complication. He has operated on or seen in consultation twelve cases of enlarged prostate treated by castration. There were two deaths, but neither could be said to be due to the operation, as one died from heart failure on the ninth day and the other from cerebral hæmorrhage on the fifth day. Two, however, nearly died of a peculiar form of traumatic delirium, which set in a day or two after the castration. Intense depression with delirium were present. Moullin considers the condition to have been simply traumatic delirium, such as sometimes occurs in feeble old people after any operation, or even after the administration of an anæsthetic, and not in any way due to the effect on the nervous system of removal of the testicle, or the absence of any substance formed by it. In two of the cases of Faulds, of Glasgow, it followed unilateral castration. These were cases in which acute mania occurred. A reference to Dr. White's table of 111 cases of castration for prostatic hypertrophy (Progress in Surgery, Hospital, November 16th, 1895) will show how rare such a condition is. In all Moullin's twelve cases but one, the improvement in micturition was definite within a week. The prostate became very considerably reduced in size, and in some the enlargement disappeared so completely that the shaft of the catheter could be felt for its whole length by the finger in the rectum. In some cases the onset of the improvement was very rapid—a few hours sometimes—and this Moullin believes to be due to rapid diminution of the congestion of the gland, as the result of removal of the testes on the nervous mechanism. In all but one case voluntary power of micturition was regained, and the residual urine fell below two ounces, cystitis disappeared, and ammoniacal urine became normal in reaction, while, in some cases, a free discharge of deposits of phosphates from the interior of the bladders occurred, often in masses of considerable size, so that pain in the urethra was experienced during their passage.

A case where marked atrophy of half of a greatly enlarged prostate followed unilateral castration occurred in the practice of Manning, of Salisbury, and is referred to by Moullin. All the patient's urinary symptoms ceased. A case is recorded by Moullin[2] where great enlargement of the middle lobe of the prostate occurred in a man in whom one testicle had completely atrophied in early life from injury to the cord. The valvular nature of the obstruction was very marked. The diagnosis was confirmed by perineal section, when a pedunculated median outgrowth nearly the size of a pigeon's egg was discovered hanging in the orifice of the bladder, and acting like a ball-valve. In cases of marked enlargement of the lateral lobes Moullin thinks unilateral castration might always be tried, for if the removal of one testicle did not prove sufficient the other could then be removed. Rovsing[3] records a case of castration for prostatic hypertrophy in a man aged 85, who although for eleven years he had not passed urine except by catheter, recovered the power of voluntary micturition, so that he was able to make water with normal frequency, and a very slight amount of residual urine remained. The result was quite unexpected, and the operation was only done to facilitate the introduction of the catheter. The case shows that over-distension, dilatation, and muscular atrophy is not always present to a degree beyond recovery in such cases. A somewhat similar case is recorded by Albanan,[4] but the time during which the patient was unable to pass urine except by catheter was only six months. The power of voluntary micturition was completely recovered and very little residual urine remained. Another case of prostatic hypertropy is recorded by Mr. Brownfield,[5] in which retention occurred, and as no instrument could be passed the bladder had to be drained above the pubes. The power of voluntary micturition did not return, and the testes were removed. The result was very good, inasmuch as he regained the power of micturition and the prostate very greatly atrophied, so that now he has no difficulty in passing water, nor has he to do so with undue frequency. The amount of residual urine, if any, is not mentioned. There are now a large number of successful cases of castration for prostatic obstruction recorded, but very few unsuccessful ones. The prostate from an unsuccessful case was shown by Mr. Davies-Colley[6] at the Pathological Society. The testes were removed from a man aged seventy-three, eight months before death. Although he was able to pass water more freely and had to do so less frequently at night, no atrophy of the prostate was observed.

Division of the vas deference instead of castration in these cases of prostatic hypertrophy is recommended by Reginald Harrison.[7] He suggests that, at any rate, it might be tried before resorting to castration. The question as to the probable benefit of excision of a portion of the vas and the result of experiments have been referred to at some length in "Surgical Progress" in The Hospital for November 16th, 1895. Mr. Harrison makes a small incision in the scrotum over the cord, isolates the vas, dissects it from the rest of the cord, and draws out a loop with a blunt hook, which he proceeds to ligature with silk and then removes. It is, he says, a very slight operation indeed, but we must not forget that castration, even when bilateral, is a very rapid operation unattended by any shock. No doubt, however, cocaine rather than general anæsthesia could more readily be employed in the former. He records two cases treated by excision of the vas. In one, phosphatic concretions were removed from the bladder at the same time that a portion of one vas was excised, and hence, although there was improvement in the condition of micturition, we can hardly put it down altogether to division of the vas. The testis and prostate are, however, said both to be undergoing atrophy, and the urethra to be diminished in length. In the other case, the details are not sufficiently given to allow us to judge of the effect of the operation. The excision was unilateral. Mansell

Moullin, in the paper already referred to,[3] mentions several cases of excision of portions of the vas for enlarged prostate. He refers to Tilden Brown's case[10] of ligature of both vasa deferentia under cocaine, in which decided benefit resulted, and to Guyon's[11] two cases, in which, without atrophy of the testes, the prostate diminished somewhat in size, and the condition of the patients was improved. Isnardi's results were more striking.[12] Very marked atrophy of the prostate followed bilateral castration in one case, and nocturnal micturition became much less frequent; in the other—a case of unilateral castration—one testicle and one side of the prostate atrophied. Rontier[13] records three cases of resection of a portion of the vas for prostatic hypertrophy, in which the results are said to be as good as after castration. He excises about an inch, after double ligature. The method, he believes, will be effectual when the prostate is soft and elastic, not when it is firm and indurated. Simple constriction of the vas by a ligature is, he believes, likely to be followed by reunion.

**Prostatectomy.**—Littlewood[14] records a case of suprapubic prostatectomy, illustrating a method of arrest of hæmorrhage by packing the bladder with a roll of cyanide gauze. A large portion of the middle of the prostate was excised, and the bleeding did not cease with the application of hot boracic lotion. The gauze practically filled the bladder. It drained away the urine at the same time that the pressure arrested the bleeding. After thirty-six hours it was removed without further hæmorrhage occurring. A method of performing prostatectomy without injury to the mucous membrane of the bladder, by removal of the gland from the perineum, is described by Alexander,[15] and two cases are recorded. The bladder is first opened above the pubes, and the prostate is pushed down into the perineum by the fingers inserted into the bladder. The membranous urethra is then opened freely on a staff, and the prostate said to be enucleated from its capsule. The bladder is drained by the opening in the membranous urethra.

[1] Lancet, Feb. 8, 1896, p. 348. [2] Ibid., Feb. 1, 1896, p. 288. [3] Centralbl. für Chir., No. 2, 1896; Epit. B. M. J., Feb 8, 1896. [4] Annales des Maladies des Organs Genito-Urinaire, No. 8, 1895; International Med. Mag., Feb., 1896. [5] Brit. Med. Journal, March 14, 1896. [6] Lancet, Feb. 8, 1896. [7] Ibid., Feb. 22, 1896. [8] Lancet, Feb. 8, 1896, p. 350. [10] New York Med. Journal, Nov. 30, 1895. [11] French Surgical Congress, 1895. [12] Gazzetta Torino, 1895, No. 31. [13] Méd. Med., No. 14, 1896; Epit. B. M. J., March 14, 1896. [14] Lancet, Jan. 4, 1896, p. 29. [15] New York Med. Journal, Feb. 8, 1896.

